# Tetraspanin 6 is a regulator of carcinogenesis in colorectal cancer

**DOI:** 10.1073/pnas.2011411118

**Published:** 2021-09-14

**Authors:** Regina Andrijes, Rahul K. Hejmadi, Matthew Pugh, Sundaresan Rajesh, Vera Novitskaya, Maha Ibrahim, Michael Overduin, Chris Tselepis, Gary W. Middleton, Balázs Győrffy, Andrew D. Beggs, Fedor Berditchevski

**Affiliations:** ^a^Institute of Cancer and Genomic Sciences, The University of Birmingham, Birmingham B15 2TT, United Kingdom;; ^b^University Hospitals Birmingham National Health Service Foundation Trust, Birmingham B15 2TH, United Kingdom;; ^c^South Egypt Cancer Institute, Assiut University, Assiut 71515, Egypt;; ^d^Department of Bioinformatics, Semmelweis University, 1094 Budapest, Hungary;; ^e^2nd Department of Paediatrics, Semmelweis University, 1094 Budapest, Hungary;; ^f^TTK Cancer Biomarker Research Group, 1117 Budapest, Hungary

**Keywords:** colorectal cancer, tetraspanin, EGFR, TGF alpha, APCmin

## Abstract

Early stages of colorectal cancer (CRC) development are characterized by a complex rewiring of transcriptional networks resulting in changes in the expression of multiple genes. Here, we demonstrate that the deletion of a poorly studied tetraspanin protein Tspan6 in Apc^min/+^ mice, a well-established model for premalignant CRC, resulted in increased incidence of adenoma formation and tumor size. We demonstrate that the effect of Tspan6 deletion results in the activation of EGF-dependent signaling pathways through increased production of the transmembrane form of TGF-α (tmTGF-α) associated with extracellular vesicles. This pathway is modulated by an adaptor protein syntenin-1, which physically links Tspan6 and tmTGF-α. In support of this, the expression of Tspan6 is frequently decreased or lost in CRC, and this correlates with poor survival. Furthermore, the analysis of samples from the epidermal growth factor receptor (EGFR)–targeting clinical trial (COIN trial) has shown that the expression of Tspan6 in CRC correlated with better patient responses to EGFR-targeted therapy involving Cetuximab. Importantly, Tspan6-positive patients with tumors in the proximal colon (right-sided) and those with KRAS mutations had a better response to Cetuximab than the patients that expressed low Tspan6 levels. These results identify Tspan6 as a regulator of CRC development and a potential predictive marker for EGFR-targeted therapies in CRC beyond RAS pathway mutations.

## Significance

Tetraspanin protein (Tspan6) is a member of the tetraspanin family. Using a combination of in vitro and in vivo assays, we demonstrate that Tspan6 functions as a tumor suppressor in colorectal cancer (CRC) by attenuating the epidermal growth factor receptor (EGFR)–based signaling axis. Tspan6 forms a tripartite complex with transmembrane form of TGF-α and an adaptor protein syntenin-1 and negatively regulates secretion of TGF-α. The expression of Tspan6 is frequently decreased in CRC, and this correlates with poor survival. Importantly, the expression of Tspan6 in CRC correlated independently of tumor molecular profile with better patient responses to Cetuximab, an EGFR-targeted therapy. These results identify Tspan6 as a regulator of CRC development and a potential predictive biomarker for EGFR-targeted therapies.

Colorectal cancer (CRC) is one of the most common malignancies worldwide which is characterized by multiple genetic and epigenetic changes. While the key genetic alterations and associated molecular pathways in CRC are well characterized, their significance in cancer development and progression remains unclear.

The expression of several proteins of the tetraspanin family have been reported to be dysregulated in CRC (1–4). Furthermore, recent RNA sequencing (RNA-seq)–based transcriptomic profiling revealed that messenger RNA (mRNA) levels for several tetraspanins were significantly altered in CRC when compared to adjacent normal tissues (5). However, the contribution of these changes to CRC initiation and progression has not been investigated. Tetraspanins are known to affect various aspects of tumor cell behavior (i.e., cell proliferation, cell migration, and invasion) by regulating key signaling pathways and thereby may influence tumor development and metastatic progression (6). Here, we used a mouse knockout model to demonstrate that the tetraspanin-6 (Tspan6), a poorly studied member of the tetraspanin family, is involved in the early stages of CRC development by regulating the epidermal growth factor receptor (EGFR) pathway. The EGFR signaling pathway plays an important role in tumor growth and the progression of CRC. Increased expression of EGFR correlates with poor prognosis in CRC, and EGFR-targeted therapies have been shown to improve survival in EGFR-positive cancer patients (7). However, a significant proportion of EGFR-positive tumors display inherent or acquired resistance to the treatment attributed to mutations in the components of the EGFR-dependent pathway (e.g., EGFR, K-/*N*-ras) or activation of alternative cancer-related pathways (8).

The biological function of Tspan6 remains uncharacterized. While an early study reported that *Tspan6* mRNA is detected in various normal epithelial tissues (9), Tspan6 knockout animals have been shown to develop normally and remain healthy over time (10). At the cellular level, Tspan6 was shown to inhibit signaling via RLR (retinoic acid-inducible gene I-like receptors) (11). We have also recently demonstrated that Tspan6 regulates the production of exosomes (10), nanovesicles that contribute to both autocrine regulation of cancer cell proliferation and paracrine communication between various cells within the tumor microenvironment (10, 12). However, the role of Tspan6 in cancer is completely unknown.

Within this study, we have utilized Tspan6 knockout animals and the Apc^min/+^ mouse model to demonstrate that Tspan6 affects EGFR-dependent signaling, thus contributing to the neoplastic transformation of intestinal and colonic epithelia. Specifically, we found that Tspan6 negatively regulates secretion of TGF-α via a molecular pathway that involves a scaffolding adaptor protein, syntenin-1, and production of extracellular vesicles. Importantly, we have established that Tspan6 expression is decreased in CRC, and the analysis of Tspan6 expression in samples from an EGFR-targeting clinical trial (the COIN study, which assessed the effect of the addition of an anti-EGFR monoclonal antibody [Cetuximab] to continuous chemotherapy on survival) revealed that that even after an adjustment for tumor-specific factors (e.g., *KRAS* mutation), Tspan6-positive patients responded better to Cetuximab-based therapies, and this correlated with improved overall survival.

## Results

### Role of Tspan6 in Intestinal Adenoma Formation.

**Fig. 1.**
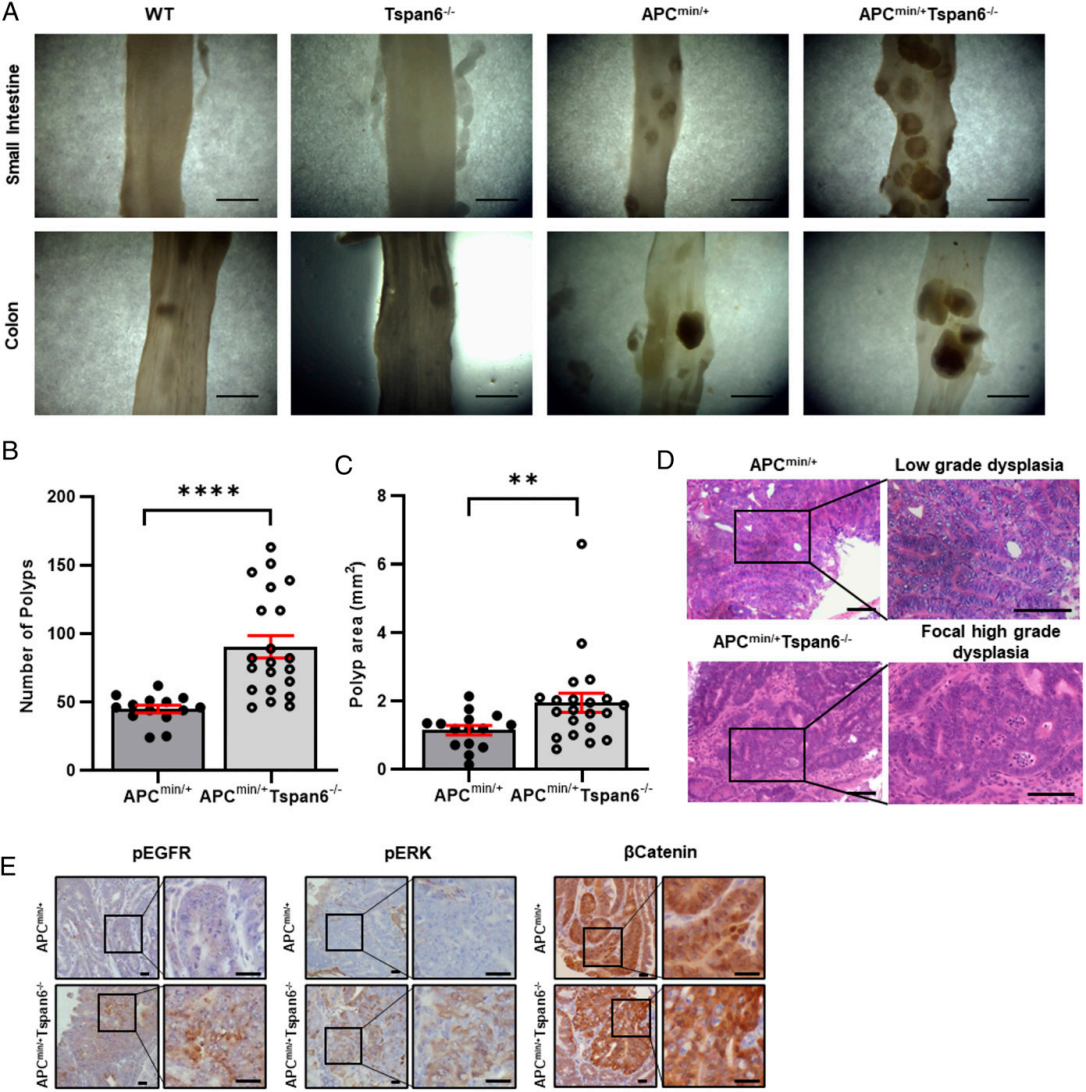
Tspan6 deficiency accentuates APC^min/+^ phenotype in vivo. (*A*) Gross appearance of small intestine and colon from wild-type, Tspan6^−/−^, APC^min/+^, and APC^min/+^Tspan6^−/−^ mice. (Scale bars: 5 mm.) (*B*) The graph represents quantifications of polyp burden in APC^min/+^ mice (*n* = 19) and APC^min/+^Tspan6^−/−^ mice (*n* = 23), showing number of polyps per analyzed mouse. Data presented as mean ± SEM, ****P < 0.0001 (Mann–Whitney *U* nonparametric test). (*C*) Distribution of intestinal polyps from APC^min/+^ (*n* = 19) and APC^min/+^Tspan6^−/−^ (*n* = 23) age-matched mice expressed as area of polyp (mm^2^). Data presented as mean ± SEM, **P = 0.0099 (Mann–Whitney *U* nonparametric test). (*D*) Representative images of hematoxylin and eosin–stained intestinal lesions in APCmin^/+^ and APC^min/+^Tspan6^−/y^ mice. Polyps of APC^min/+^ present with low-grade dysplastic adenomas throughout the small bowel. Loss of Tspan6 in APC^min/+^ mice results in the formation of lesions with focal high-grade malignant changes of intestinal mucosa. (Scale bars: 50 µm.) (*E*) Tspan6 deficiency results in hyper-activated MAPK signaling pathway. Representative images of pEGFR, pERK, and β-Catenin expression in intestinal polyps of APC^min/+^ (*n* = 5) and APC^min/+^Tspan6^−/−^ mice (*n* = 5). (Scale bars: 20 µm.)

We noted that Tspan6 was listed among genes whose expression is significantly decreased in cancer-associated polyps as compared to cancer-free polyps (13). To explore the significance of this observation and examine the involvement of Tspan6 in CRC, we first investigated whether genetic deletion of Tspan6 affects gastrointestinal tissues (GIT) in mice. Tspan6 knockout animals developed normally, and 1-y-old mice display no apparent macroscopic abnormalities of their internal organs (10). To examine the involvement of Tspan6 in the neoplastic transformation of gastrointestinal tissues, we crossed Tspan6 knockout (Tspan6^−/−^) animals with APC^min/+^ mice, a well-characterized model for colorectal cancer. The deletion of Tspan6 on the APC^min/+^ background had no apparent effect on embryonic and postnatal development with animals born at the expected Mendelian ratio and appeared physiologically normal (*SI Appendix*, Table S1). As expected, by the age of 14 to 18 wk, APC^min/+^ mice developed polyps in the small intestine and, occasionally, in the large intestine (Fig. 1 *A–D*). Notably, the number of polyps in the aged-matched APC^min/+^/Tspan6^−/−^ animals was higher, with the mean number of 45 and 90 polyps per mouse in APC^min/+^ and APC^min/+^Tspan6^−/−^ mice, respectively, *P* < 0.0001 (Fig. 1 *A* and *B*). Furthermore, we observed an increase in the average size of polyps in APC^min/+^/Tspan6^−/−^ mice when compared to the APC^min/+^ animals, with the mean size 1.144 and 1.957 mm^2^ in APC^min/+^ and APC^min/+^Tspan6^−/−^ mice, respectively, *P* = 0.0099 (Fig. 1*C*). Histological analysis indicated that all lesions in APC^min/+^ and APC^min/+^/Tspan6^−/−^ mice could be classified as adenomas (Fig. 1*D*). When analyzed in more detail, there was a substantial increase in the incidence of high-grade dysplasia in APC^min/+^/Tspan6^−/−^ mice as compared for APC^min/+^ animals (43% versus 5%, respectively, Table 1). Taken together, these results suggest that the loss of Tspan6 contributes to early stages of intestinal carcinogenesis.

**Table 1.** Incidence of low-grade and focal high-grade adenomas in small intestine and colon of APC^min/+^ (*n* = 19) and APC^min/+^Tspan6^−/−^ (*n* = 23) mice

Tumor location	Tumor grade (dysplasia)	APC^min/+^ (*n* = 19)	APC^min/+^ Tspan6^−/−^ (*n* = 23)
Small intestine	Low-grade	18	20
	High-grade (focal)	1	10
Colon	Low-grade	3	15
	High-grade (focal)	0	9

### Tspan6 Affects Autocrine Signaling in Intestinal Epithelial Cells.

To identify molecular pathways associated with Tspan6, we compared RNA expression profiles in tumors formed in APC^min/+^ and APC^min/+^/Tspan6^−/−^ mice (Table 2). Pathway analysis demonstrated that the deletion of Tspan6 resulted in the enrichment of mRNAs encoding proteins involved in Wnt and MAPK pathways (*P* = 0.016). While further immunohistological analysis demonstrated comparable expression and tissue distribution of β-catenin in APC^min/+^ and APC^min/+^Tspan6^−/−^ polyps, activation of EGFR and Erk1/2 in APC^min/+^Tspan6^−/−^ polyps was notably stronger than that seen in polyps of APC^min/+^ animals (Fig. 1*E*). Therefore, we chose to specifically focus on the EGFR-MAPK pathway.

**Table 2.** Kyoto Encyclopedia of Genes and Genomes pathway analysis of differentially expressed genes in intestinal polyps of APC^min/+^ (*n* = 3) and APC^min/+^Tspan6^−/−^ mice (*n* = 5)

Kyoto Encyclopedia of Genes and Genomes pathway	*P* Value
MAPK signaling pathway	0.003212
Ubiquitin-mediated proteolysis	7.81E-07
Endocytosis	0.011859
Focal adhesion	0.019936
Chemokine signaling pathway	0.019289
Wnt signaling pathway	0.016458
Neurotrophin signaling pathway	0.02865
Insulin signaling pathway	0.042612
T cell receptor signaling pathway	0.033175
Phosphatidylinositol signaling system	0.011894
ErbB signaling pathway	0.028932
Nucleotide excision repair	0.019021

Pathways with enrichment *P* < 0.05 were considered significant.

**Fig. 2.**
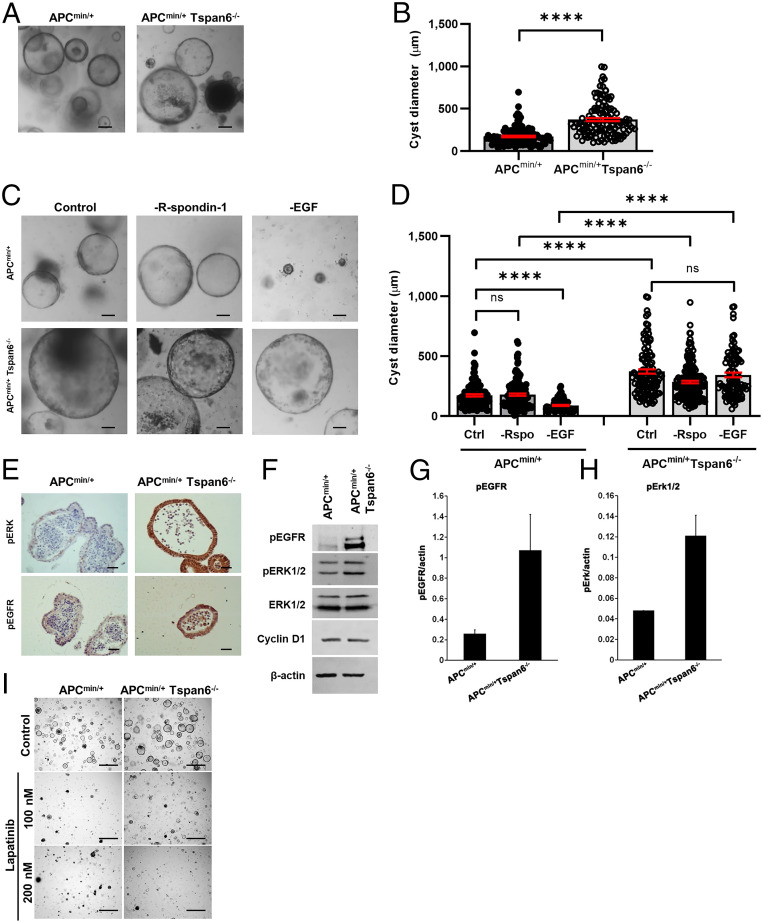
Tspan6 loss results in EGF-independent growth of intestinal organoids derived from APC^min/+^ and APC^min/+^Tspan6^−/−^ mice. (*A*) Representative pictures of the APC^min/+^ and APC^min/+^Tspan6^−/−^ intestinal organoids (*n* = 3). Mouse intestinal organoids were derived from APC^min/+^ and APC^min/+^Tspan6^−/−^ mice and cultured in mouse intestinal organoid media for 5 d. (Scale bars: 100 μm.) (*B*) Quantification of size distribution of intestinal organoids from APC^min/+^ (*n* = 116) and APC^min/+^ Tspan6^−/−^(*n* = 119) mice. The measurement of diameter of each organoid was carried out using ImageJ; at least 10 fields of view were analyzed (*n* = 3). Data presented as mean ± SEM, ****P < 0.0001; ns, not significant (Mann–Whitney *U* nonparametric test). (*C*) Representative pictures of the APC^min/+^ and APC^min/+^Tspan6^−/−^ intestinal organoids cultured in complete organoid growth media containing EGF, Noggin, and R-spondin-1 (control) and in media lacking R-spondin-1 (−R-spondin-1) or EGF (−EGF) (*n* = 3). (Scale bars: 100 μm.) (*D*) Quantification of size distribution of intestinal organoids from APC^min/+^ and APC^min/+^Tspan6^−/−^ mice cultured in complete growth media (Ctrl) (*n* = 116 and *n* = 119, respectively) in media lacking R-spondin-1 (−Rspo) (*n* = 144 and *n* = 195, respectively) or EGF (−EGF) (*n* = 95 and *n* = 94, respectively). The measurement of diameter of each organoid was performed using ImageJ; at least 10 fields of view were analyzed (*n* = 2). Data presented as mean ± SEM, ****P < 0.0001; ns, not significant (one-way ANOVA test). (*E*) Representative images of pEGFR and pERK IHC staining of FFP-embedded APC^min/+^ and APC^min/+^Tspan6^−/−^ mouse intestinal organoids (*n* = 5). (Scale bars: 25 μm.) (*F*) Western blot showing the increase in pEGFR and pERK expression in APC^min/+^ and APC^min/+^Tspan6^−/−^ mouse intestinal organoids. (*G*) Quantification of EGFR activation relative to β-actin expression in APC^min/+^ and APC^min/+^Tspan6^−/−^ mouse intestinal organoids. Data presented as mean of two independent experiments ± SEM. (*H*) Quantification of Erk1/2 activation relative to β-actin expression in APC^min/+^ and APC^min/+^Tspan6^−/−^ mouse intestinal organoids. Data presented as mean of two independent experiments ± SEM. (*I*) Representative images of APC^min/+^ and APC^min/+^Tspan6^−/−^ organoids in response to pan-EGFR inhibitor lapatinib after 5 d of culture. (Scale bars: 500 μm.)

To explore these Tspan6-dependent differences in molecular pathways in intestinal epithelial cells in more detail, we established intestinal organoid cultures from the APC^min/+^ and APC^min/+^/Tspan6^−/−^ animals. Both APC^min/+^ and APC^min/+^/Tspan6^−/−^ organoids formed hollow cysts when grown in a standard growth media supplemented with EGF, Noggin, and R-spondin-1 (ENR). Further quantitative analysis demonstrated that APC^min/+^/Tspan6^−/−^ organoids were larger in size when compared to APC^min/+^ organoids (Fig. 2 *A* and *B*). To examine whether the Wnt and MAPK pathways contributed to these Tspan6-dependent differences in organoid growth, we cultured organoids in media lacking R-spondin-1 or EGF. While both APC^min/+^ and APC^min/+^/Tspan6^−/−^ organoids could grow in media lacking R-spondin-1, the withdrawal of EGF significantly reduced the size of APC^min/+^ organoids with only a minimal effect on the growth of APC^min/+^/Tspan6^−/−^ organoids (Fig. 2 *C* and *D*). These results demonstrated that the deletion of Tspan6 is leading to intrinsic activation of the EGF-dependent signaling pathway. Accordingly, we observed increased activation of pErk1/2 in APC^min/+^/Tspan6^−/−^ organoids (Fig. 2 *E* [immunohistochemistry (IHC)], *F*, and *H* [Western blotting]). Importantly, we found that the level of EGFR activation was also higher in APC^min/+^/Tspan6^−/−^ organoids (Fig. 2 *E–G*), thus suggesting that Tspan6 regulates early events in the receptor activation. Furthermore, the activation of EGFR was critical for the growth of APC^min/+^/Tspan6^−/−^ organoids, as it was strongly suppressed by lapatinib, a specific EGFR inhibitor (Fig. 2*I* and *SI Appendix*, Fig. S1).

We further confirmed a functional link between Tspan6 and EGFR signaling using Caco-2 cells. These cells form colonies with a central lumen (often referred to as cysts) when cultured for an extended period of time in the three-dimensional extracellular matrix (3D ECM) (14). We found that the proportion of colonies with a central lumen increased by approximately threefold when CaCo-2 cells were cultured in standard growth media supplemented with Cetuximab (humanized anti-EGFR antibodies, which prevent binding of EGFR ligands to the receptor) (*SI Appendix*, Fig. S2*A*). These results suggested that blocking autocrine EGFR signaling facilitates lumen formation. Importantly, ectopic expression of Tspan6 in Caco-2 cells, which express low levels of the endogenous protein, increased the proportion of colonies with a central lumen (*SI Appendix*, Fig. S2*B*), suggesting that the expression of Tspan6 suppresses autocrine activation of EGFR. Accordingly, the EGFR activation in the control CaCo-2 cells (CaCo-2/pLVx) cultured in 3D ECM was significantly higher than in CaCo-2/Tspan6 cells (*SI Appendix*, Fig. S2*C*).

**Fig. 3.**
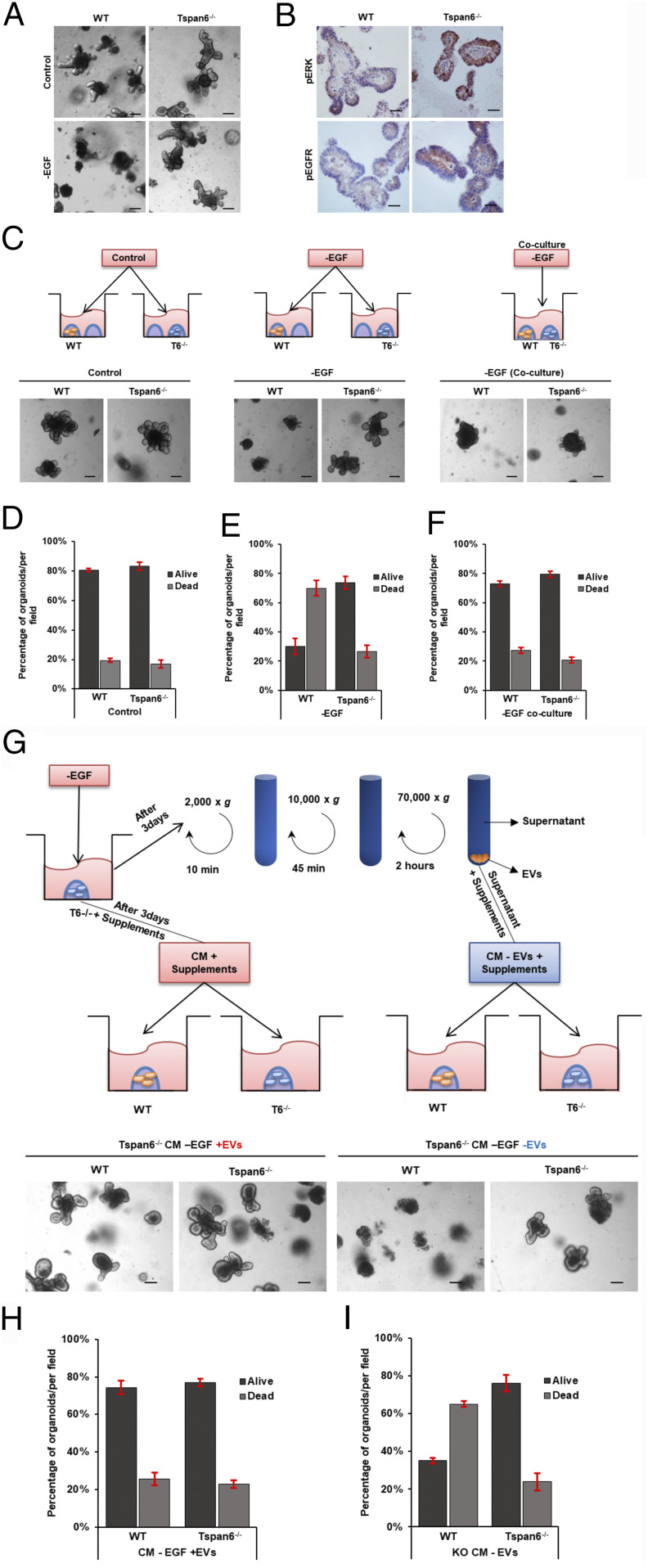
Tspan6 negatively regulates secretion of an EGFR ligand in mouse intestinal organoids. (*A*) Representative images of wild-type (WT) and Tspan6^−/−^ organoids cultured in complete organoid growth media containing EGF, Noggin, and R-spondin-1 (control) and in media lacking EGF (−EGF) (*n* = 3). (Scale bars: 100 μm.) (*B*) Representative images of pEGFR and pERK IHC staining of FFP-embedded WT and Tspan6^−/−^ mouse intestinal organoids (*n* = 5). (Scale bars: 25 μm.) (*C*) Schematic diagram outlining the experimental setting to examine the effect of an extracellular EGFR ligand secreted by Tspan6^−/−^ organoids on growth. WT and Tspan6^−/−^ organoids were cultured in separate wells in complete growth media (control) and media lacking EGF (−EGF) or cocultured in the same well in media lacking EGF (coculture-EGF) for 5 d. (*Lower*) Representative pictures of the indicated treatments. Coculturing with Tspan6^−/−^ organoids rescued WT organoids in –EGF conditions (*n* = 3). (Scale bars: 100 μm.) (*D–F*) Quantification of live and dead organoids per field of view cultured in (*D*) complete growth media (Control), (*E*) media lacking EGF (−EGF), and (*F*) cocultured in the same well in media lacking EGF (coculture-EGF). Data presented as mean ± SEM, *n* = 3. (*G*) Schematic diagram showing the experimental setting to examine the contribution of EVs secreted by Tspan6^−/−^ organoids on growth. Media conditioned (CM) by Tspan6^−/−^ organoids in EGF-free conditions was collected after 3 d of growth. Media was depleted of EVs by differential centrifugation in three steps followed by supplementing with essential organoid media components (without growth factors) and used to culture WT organoids (−EVs). Nonprocessed conditioned media was also supplemented with essential organoid media components and used to culture WT organoids as control (+EVs). (*Lower*) Representative pictures of the indicated treatments. Culturing WT organoids in Tspan6^−/−^ CM nondepleted of EVs supported growth of WT organoids, and depletion of EVs in media resulted in cellular death of WT organoids (*n* = 3). (Scale bars: 100 μm.) (*H* and *I*) Quantification of live and dead organoids per field of view cultured in nondepleted (*H*) or depleted of EVs media (*I*), which was conditioned (CM) by Tspan6^−/−^ organoids in EGF-free conditions. Data presented as mean ± SEM, *n* = 3.

There exists an extensive crosstalk between Wnt- and EGFR-dependent signaling pathways in colorectal cancer (15). To examine whether alterations in Wnt signaling (i.e., *APC* mutation) affects Tspan6-dependent changes in EGFR activation, we repeated the EGF withdrawal experiments using intestinal organoids derived from the Tspan6 knockout mice. Similar to the results involving APC^min/+^/Tspan6^−/−^ organoids (Fig. 2*D*), the withdrawal of EGF had no notable effect on the growth and differentiation of organoids derived from the Tspan6-deficient animals (control wild-type and APC^min/+^ organoids died by apoptosis in the absence of EGF) (Fig. 3*A* and *SI Appendix*, Fig. S3). Accordingly, the activation of EGFR and Erk1/2 was also increased in Tspan6^−/−^ organoids when compared to control (Fig. 3*B*). Furthermore, the growth of both Tspan6^−/−^ and control organoids in complete media was also suppressed by lapatinib (*SI Appendix*, Fig. S4). These results further support the idea that Tspan6 directly affects EGFR-dependent signaling. To investigate the underlying molecular pathways in more detail, we established coculture experiments in which control Tspan6-expressing organoids were grown in EGF-depleted media in the presence of Tspan6^−/−^ organoids (plated in the same well). These experiments demonstrated that the negative effects of EGF depletion on the growth of Tspan6-expressing organoids could be reversed when cocultured with Tspan6^−/−^organoids (Fig. 3*C*), thus suggesting that Tspan6 negatively regulates the autocrine pathway of EGFR signaling.

### Autocrine Regulation of Organoid Growth Is Mediated by Extracellular Vesicles.

Autocrine signaling can be mediated by secreted factors or involve cell-derived vesicles [e.g., exosomes may carry both EGFR ligands, such as TGF-α and amphiregulin (AREG) (16)]. Thus, we examined whether a transstimulating proliferative effect of Tspan6^−/−^ organoids under EGF-free conditions is retained when media is depleted of extracellular vesicles (EVs) (Fig. 3*G*). Strikingly, EV-depleted media could not support proliferation and viability of Tspan6-expressing organoids which rapidly undergo cell death after 3 to 5 d of culturing. By contrast, Tspan6^−/−^organoids were able to grow and differentiate (Fig. 3 *G* and *H*).

### The Role of TGF-α in Tspan6-Dependent Autocrine Regulation of Organoid Growth.

**Fig. 4.**
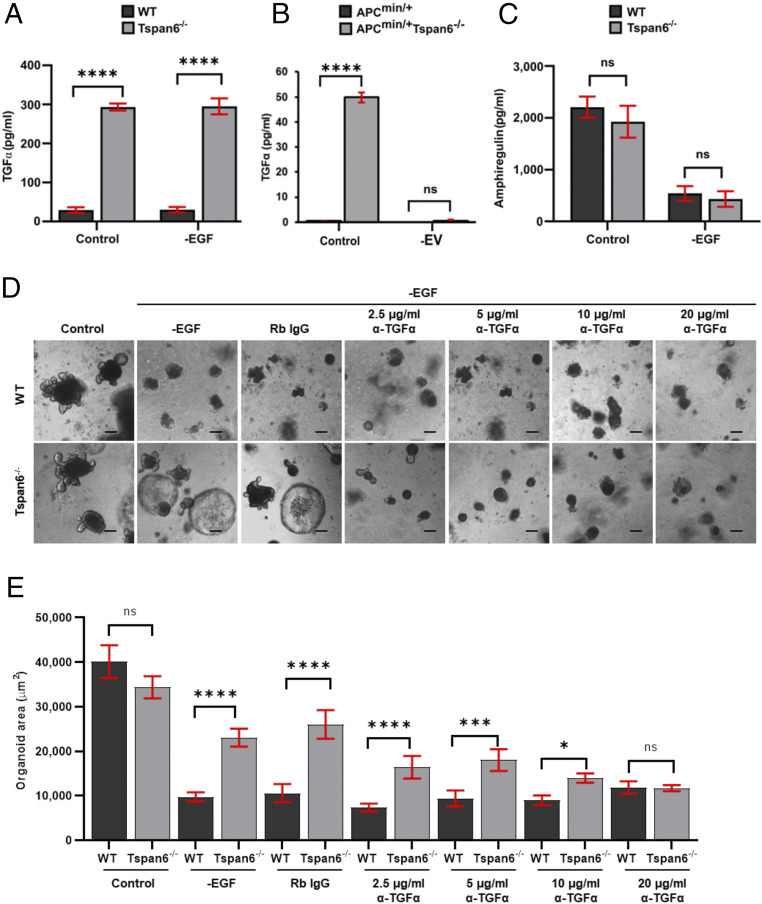
Tspan6 negatively regulates secretion of TGF-α. (*A*) Enzyme-linked immunosorbent assay (ELISA) analysis of TGF-α present in media collected from wild-type (WT) and Tspan6^−/−^ intestinal organoids after culturing in complete growth media (control) or in EGF-free media (−EGF) for 5 d (*n* = 2). Data presented as mean ± SEM, ****P < 0.0001, ns, not significant (two-way ANOVA test). (*B*) ELISA analysis of TGF-α in media collected from APC^min/+^ and APC^min/+^Tspan6^−/−^ intestinal organoids after depletion of EVs (*n* = 2). Data presented as mean ± SEM, ****P < 0.0001, ns, not significant (two-way ANOVA test). (*C*) ELISA analysis of amphiregulin present in media collected from WT and Tspan6^−/−^ intestinal organoids after 5 d of culture. Data presented as mean ± SEM, ****P < 0.0001, ns, not significant (*n* = 3) (two-way ANOVA test). (*D*) Representative images of WT and Tspan6^−/−^ organoids cultured in control media in media lacking EGF (−EGF) supplemented with rabbit IgG (Rb IgG) or increasing concentrations of anti-mouse TGF-α antibody (α-TGF-α) (2.5 μg/mL – 20 μg/mL). (Scale bars: 100 µm.) (*E*) Distribution of organoid size cultured in –EGF conditions in the presence or absence of anti–TGF-α neutralizing antibody. The organoid size was measured using ImageJ and presented as area in square micrometers. For statistical analysis for each condition, 10 fields were captured and >50 organoids were analyzed. Data presented as mean ± SEM, ****P < 0.0001, ***P = 0.0003, *P = 0.0166, ns, not significant (one-way ANOVA test).

The transmembrane form of the TGF-α and AREG have been shown to associate with EVs (16) and therefore may contribute to the integrity and survival of intestinal organoids under EGF-free culturing conditions. Thus, we compared the levels of TGF-α and AREG in media conditioned by the control Tspan6-expressing and Tspan6^−/−^ organoids. These experiments demonstrated that Tspan6^−/−^ organoids secreted ∼10-fold higher levels of TGF-α: 293.1 ± 15.5 pg/mL for Tspan6^−/−^ organoids compared with 23.3 ± 12.7 pg/mL for Tspan6-expressing organoids (Fig. 4*A*). The difference in the levels of TGF-α was even more dramatic when we compared media conditioned by APC^min/+^ and APC^min/+^/Tspan6^−/−^ organoids—0.09 ± 0.01 pg/mL and 49.8 ± 0.4 pg/mL, respectively. Importantly, the removal of EVs from the conditioned media dramatically reduces the amount of TGF-α in the media (below 0.05 pg/mL) (Fig. 4*B*). These results strongly suggest that TGF-α is secreted as the EV-associated transmembrane protein. By contrast, there was no difference in the level of AREG in media conditioned by Tspan6-expressing and Tspan6-negative organoids (Fig. 4*C*). To prove directly the involvement of tmTGF-α in autocrine-dependent growth regulation of Tspan6^−/−^ organoids, Tspan6^−/−^ organoids were cultured in EGF-depleted growth media supplemented with anti-TGF-α blocking antibodies. The presence of the antibodies suppressed growth and differentiation of Tspan6^−/−^ organoids in a concentration-dependent manner (Fig. 4 *D* and *E*).

The nanoparticle tracking analysis was used to examine whether Tspan6 deletion affected the quantity and size of vesicles secreted by organoids cultured in EGF-depleted media. These experiments demonstrated that media conditioned by the control and Tspan6^−/−^organoids have comparable concentrations of the EVs and size distribution (*SI Appendix*, Fig. S5*A*). Furthermore, we found that deletion of Tspan6 or overexpression of the protein did not affect cellular levels of syntenin-1, a key protein in production of exosomes (17) (*SI Appendix*, Fig. S5 *B–E*).

### Tspan6 Is Associated with TGF-α Via Syntenin-1.

**Fig. 5.**
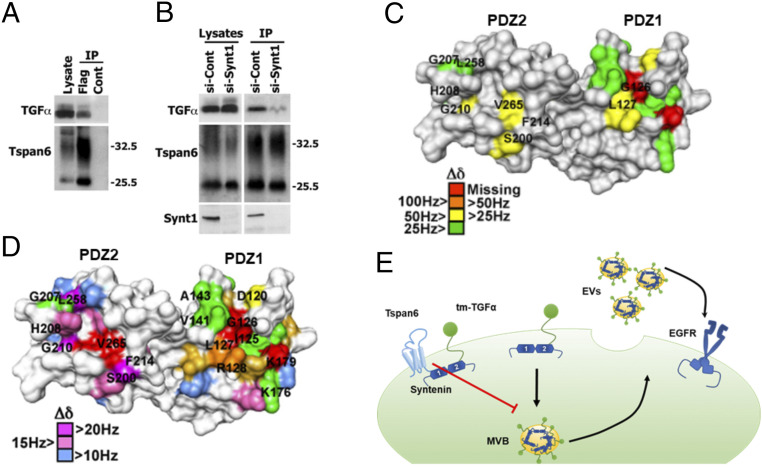
Tspan6 is associated with transmembrane form of TGF-α (tmTGF-α). (*A*) Coimmunoprecipitation (IP) of Tspan6 and tmTGF-α. IP studies were carried out with lysates prepared from HEK293T cells expressing the Flag-Tspan6 (Flag) or empty vector (control). Flag-Tspan6 protein was immunoprecipitated with Flag-agarose beads and then immunoblotted using a polyclonal Tspan6 antibody and polyclonal TGF-α antibody. (*B*) IP of Tspan6 and TGF-α in cells after syntenin-1 depletion. IP studies were carried out with lysates prepared from HEK293T cells expressing the Flag-Tspan6 (Flag) or empty vector (control) treated with syntenin-1 small interfering (siRNA) (si-synt1) or nontargeting siRNA (si-cont). (*C*) NMR analysis of syntenin-1 interaction with the C-terminal peptide of tmTGF-α. Syntenin-1 PDZ domain residues involved in binding to the C-terminal peptide of tmTGF-α, based on NMR chemical shift perturbation, are indicated on the surface of syntenin-1 PDZ1-2 monomer (1N99.pdb). The residues displaying chemical shift changes (Δδ) greater than the mean value plus one (55 Hz) are color-coded as indicated. (*D*) NMR analysis of syntenin-1 interaction with C-terminal peptides of Tspan6 and tmTGF-α. Syntenin-1 PDZ domain residues involved in binding to the C-terminal peptide of Tspan6 (*Top*), based on NMR chemical shift perturbation, are indicated on the surface of syntenin-1 PDZ1-2 monomer (1N99.pdb). The residues displaying combined chemical shift changes (Δδ) greater than the mean value plus one (55 Hz) are color-coded as indicated. Syntenin-1 residues displaying Δδ greater than 25 Hz upon interaction with a mixture of Tspan6 and tmTGF-α peptides are indicated (*Bottom*), with red and green indicating proximal and distal, respectively, to the C-terminal peptide–binding pocket. (*E*) Proposed model describing how Tspan6 regulates activation of EGFR-dependent by regulating secretion of EV-associated tmTGF-α. When expressed, Tspan6 forms a molecular complex with tmTGF-α via syntenin-1, thus inhibiting the recruitment of tmTGF-α into multivesicular bodies (MVBs) and subsequent secretion into extracellular space. The loss of Tspan6 favors syntenin-1–mediated recruitment of tmTGF-α into EVs and subsequent activation of EGFR.

We next examined whether the Tspan6–syntenin-1 complex controls cellular distribution of the tmTGF-α. Not only could Tspan6 and tmTGF-α be coimmunoprecipitated (Fig. 5*A* and *SI Appendix*, Fig. S6), but the interaction between the proteins was significantly reduced in cells depleted of syntenin-1 (Fig. 5 *A* and *B* and *SI Appendix*, Fig. S6). Furthermore, NMR titration analysis of ^15^*N*-labeled syntenin-1 PDZ1-2 with unlabeled tmTGF-α and Tspan6 peptides corresponding to the C termini of the proteins demonstrated that syntenin-1 can simultaneously bind peptides corresponding to the C-terminal cytoplasmic portion of Tspan6 and tmTGF-α (Fig. 5*D*). Taken together, these results suggest that Tspan6 in complex with syntenin-1 controls cellular distribution of tmTGF-α so that the decrease (or loss) of Tspan6 will shift the balance toward recruitment of tmTGF-α to EVs (Fig. 5*E*).

To examine the functional significance of the Tspan6–syntenin-1 interaction, we studied the effect of syntenin-1 depletion on the formation of the central lumen by Caco-2/Tspan6 cells cultured in 3D ECM. We observed a dramatic reduction in the number of colonies with a central lumen when CaCo-2/Tspan6 cells were depleted of syntenin-1 (*SI Appendix*, Fig. S7). These data suggest that syntenin-1 plays a critical role in the Tspan6-dependent autocrine regulation of EGFR activation (*SI Appendix*, Fig. S2).

### Tspan6 Is a Predictive Marker for Cetuximab-Based Chemotherapy in CRC Patients.

**Fig. 6.**
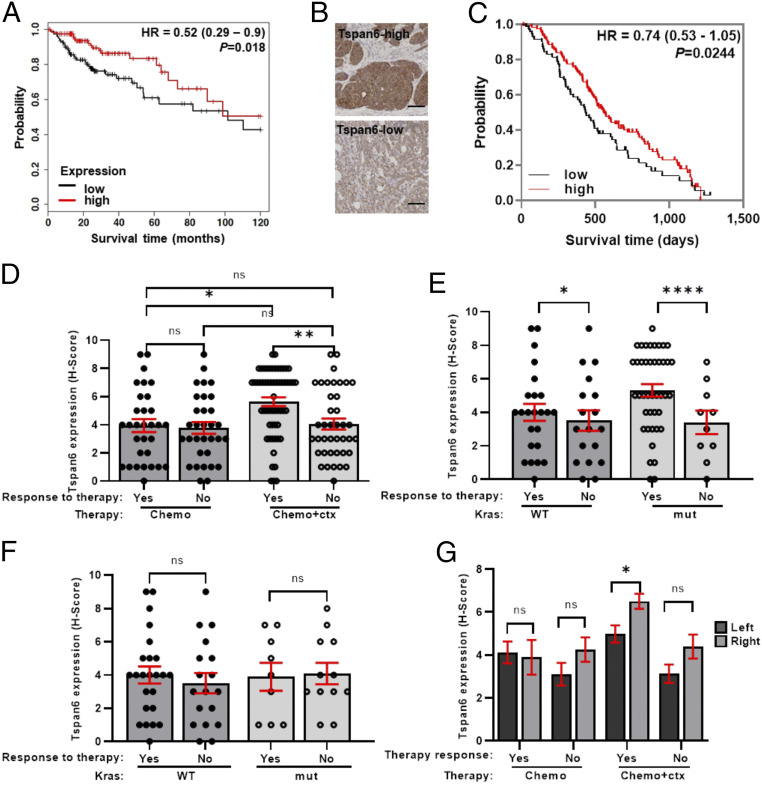
The decreased expression of Tspan6 correlates with poor survival prognosis in patients with colorectal adenocarcinomas. (*A*) Kaplan–Meier survival curves for 10-y overall survival of CRC patients with Tspan6-high and Tspan6-low expression of CRC patients with adenocarcinomas (*n* = 252). *P* values were determined by log-rank test and are shown for comparisons of Tspan6-high expression (*n* = 125) and Tspan6-low expression (*n* = 127) in adenocarcinomas. (*B*) Representative images of Tspan6-high and Tspan6-low protein expression in CRC tumors from patients of COIN clinical trial cohort. (*C*) Kaplan–Meier survival curves for overall survival of CRC patients with Tspan6-high and Tspan6-low expression of the COIN cohort (*n* = 182). *P* value was determined by Gehan–Breslow–Wilcoxon test and are shown for comparisons of Tspan6-high (*n* = 112) and Tspan6-low (*n* = 70) expression in the whole cohort. (*D–G*) The correlation of Tspan6 expression with responses of CRC patients treated with chemotherapy alone or chemotherapy in combination with Cetuximab (COIN study). (*D*) Responses in the whole cohort of patients treated with chemotherapy (response *n* = 31, no response *n* = 31) and patients treated with chemotherapy combined with cetuximab (response *n* = 40, no response *n* = 58). Data presented as mean ± SEM, **P = 0.0043, *P = 0.0106, ns: not significant (two-way ANOVA test). (*E*) Responses of the patients with mutated K-Ras (*n* = 46) or WT K-Ras (*n* = 52) to the chemo + Cetuximab therapy. Data presented as mean ± SEM, ****P < 0.0001, *P = 0.0415, ns: not significant (two-way ANOVA test). (*F*) Responses of the patients with mutated K-Ras (*n* = 21) or WT K-Ras (*n* = 42) to the chemotherapy only. Data presented as mean ± SEM, ns: not significant (two-way ANOVA test). (*G*) The expression of Tspan6 in tumors that have responded to the therapy (chemotherapy only or chemotherapy combined with cetuximab) in left-sided (*n* = 111) or right-sided (*n* = 72) colorectal adenocarcinomas. Data presented as mean ± SEM, *P = 0.0125 (two-way ANOVA test).

We used 463 colorectal tumor samples from the Cancer Genome Atlas dataset [the largest sequenced and publically available dataset of CRC samples (18)] to correlate expression of *TSPAN6* with survival. In this analysis, we found that the survival of patients with high *TSPAN6*-expressing adenocarcinomas was significantly better compared to patients with low *TSPAN6*-expressing adenocarcinomas (Fig. 6*A*). We also found that the expression of *TSPAN6* correlates with better survival of patients with advanced tumors (T4, N2, or M1 stages) and tumors with extensive lymphovascular invasion (*SI Appendix*, Fig. S8).

To extend our findings linking Tspan6 and EGFR-dependent signaling in colorectal cancer, we investigated whether the expression of Tspan6 can predict a response to the EGFR-targeting therapies in vivo. Thus, the expression of Tspan6 was analyzed by immunohistochemistry in 184 tumor samples from the COIN study (19). The COIN study assessed the effect of the addition of an anti-EGFR monoclonal antibody (Cetuximab) to continuous chemotherapy on the survival in patients with metastatic colorectal cancer. In agreement with our gene expression analysis, patients expressing Tspan6 had better prognosis than those with low or no expression of the protein (Fig. 6 *B* and *C*). Furthermore, we found that Tspan6 expression predicted better responses to the Cetuximab-based therapy (Fig. 6*D*). Notably, we observed that patients with mutated K-ras who did respond to the cetuximab-based therapy expressed higher levels of Tspan6 when compared to those who were treatment resistant (Fig. 6*E*). Furthermore, patients with Tspan6 expressing right-sided CRCs responded better to the cetuximab-based therapy as compared to left-sided tumors (Fig. 6*G*). By contrast, the expression of Tspan6 had no prognostic or predictive value for patients from the pure chemotherapy arm (i.e., no addition of Cetuximab) (Fig. 6*F*).

To further understand the relationship between Tspan6 expression and response to treatment in the COIN trial, we carried out reverse stepwise logistic regression with response at 12 wk (response/no response) or overall response (response/no response) as independent variables, with Tspan6 expression, KRAS/BRAF/NRAS/PI3KCA mutation status, microsatellite instability status, age, gender, and tumor site (coded as left/right) as dependent variables. For both the 12-wk tumor response (odds ratio [OR] 1.43, 95% CI 1.17 to 1.76, *P* = 0.001, model area under the curve [AUC] = 0.80) and overall response (OR 1.66, 95% CI 1.30 to 2.12, *P* < 0.001, model AUC = 0.86), higher Tspan6 expression independently predicted for the response even after adjustment, suggesting that it has utility for stratifying patients who will respond to anti-EGFR therapy.

## Discussion

Development and metastatic progression in CRC rely on intrinsic genetic/epigenetic changes in intestinal epithelium that control a complex network of signaling pathways involving various cell types in the tumor microenvironment. Within this study, we demonstrate that Tspan6 suppresses early stages of CRC development. Mechanistically, we found that Tspan6 functions as a regulator of EGFR signaling by suppressing an autocrine-dependent pathway involving the secretion of TGF-α via EVs. Importantly, we demonstrated that the expression of Tspan6 is associated with improved responses to EGFR-targeted therapies and prolonged patient survival.

Despite a clear benefit of EGFR-targeted therapies for metastatic CRC, primary and acquired resistance remains a significant challenge and poses limitations in their application (20). Oncogenic K-ras/N-ras mutations along with mutations in genes encoding other proteins downstream of the EGFR signaling pathways (e.g., *BRAF*, *PI3KCA*) have been identified as negative predictive biomarkers. Here, we used samples from the COIN clinical trial to demonstrate that the expression of Tspan6 predicts a positive response to Cetuximab-based therapies irrespective of K-ras/N-ras mutations. Our data indicate that the predictive power of Tspan6 expression is linked to the key role played by this protein in modifying the biological activity of EVs secreted by cancer cells. Earlier studies suggested that the increased expression of EGFR ligands can promote resistance to anti-EGFR monoclonal antibody (21, 22). Thus, we propose that Tspan6-dependent sensitization to the EGFR-targeted therapy is due to the suppression of TGF-α secretion by cancer cells. Conversely, the increased secretion of TGF-α in patients with low or no expression of Tspan6 is likely to decrease the suppressive effect of Cetuximab. This model may also explain why Tspan6-expressing right-sided CRC in the COIN cohort responded to the cetuximab-based therapy. Previous studies demonstrated that the right-sided tumors express higher levels of EGFR (23) and, consequently, were less sensitive to the cetuximab-based treatments (24). Our data suggest that the increased expression of EGFR in those tumors will be responsive to the treatment if cancer cells express Tspan6, which suppresses secretion of TGF-α. Thus, Tspan6 represents the first example of a positive predictive biomarker for the responsiveness of right-sided CRC patients to Cetuximab-based therapies.

Although we demonstrate that the loss of Tspan6 activates the TGF-α–EGFR signaling axis in the intestinal epithelium, this is not sufficient to drive the malignant transformation. Similarly, Bilger and colleagues reported that the increased expression of TGF-α transgene in Apc^min/+^ mice did not result in the malignant phenotype (25). Taken together, these results suggest that additional genetic/epigenetic changes are required to drive malignant transformation in the context of the Apc^min/+^ genetic model. It is also possible that the contribution of the TGF-α−EGFR signaling pathway (and, therefore, Tspan6) to the development of the malignant phenotype involves other cell types in the tumor microenvironment (26). The suppression of EGFR-dependent signaling in nonepithelial cells in Tspan6-positive CRC and attenuation of ensued paracrine signaling acting on epithelial cells may explain why responses to Cetuximab-based therapy was independent of the Ras mutation status in this group of CRC patients.

Our data indicate that Tspan6 plays an important role in defining the composition of EVs. In this regard, we demonstrated that two tandemly placed PDZ domains of syntenin-1, the only known molecular partner of Tspan6, can physically link Tspan6 with tmTGF-α. Accordingly, we predict that Tspan6 and syntenin-1 act together to direct the intracellular distribution of tmTGF-α so that the Tspan6–syntenin-1 complex would prevent the recruitment of tmTGF-α to EVs. Conversely, down-regulation (or loss) of Tspan6 in colorectal cancers results in the redistribution of the tmTGF-α−syntenin-1 complex to the EVs. As syntenin-1 also functions as a scaffolding adaptor for other transmembrane proteins (27), one would expect that Tspan6 has a wider pleiotropic effect on paracrine communication involving Tspan6-expressing cells. Further delineation of the molecular composition of the Tspan6-syntenin-1–centered protein network will provide a more comprehensive view as to how Tspan6 can affect the tumor microenvironment in CRC and other types of cancer.

While our results strongly suggest that Tspan6-dependent changes in the TGF-α−EGFR signaling axis affect the premalignant transformation of intestinal epithelium, we cannot exclude that other Tspan6-associated pathways (Table 2) have a role in the later stages of CRC development. Hence, further work will be necessary to investigate possible contributions of these or other changes that are known to associate with tetraspanin proteins (e.g., activation of other receptor tyrosine kinases) to intestinal tumorigenesis.

## Materials and Methods

### Clinical Data and Patient Information.

Tumor samples and medical records from S-CORT and COIN colorectal cancer clinical trials were obtained from the Human Biomaterials Resource Centre, University of Birmingham under the local ethics committee approval (ref no.16–250).

### Mice.

C57BL/6 wild-type and APC^min/+^ mice were purchased from Jackson Laboratory. Tspan6^−/−^ mice were purchased from VelociGene pharmaceutical company. Tspan6^−/−^ C57BL/6 mice (10) were crossed with APC^min/+^ mice and bred in our laboratory. The gene phenotype was routinely confirmed. All animal procedures were performed according to Home Office guidelines (United Kingdom) and the UK Animals (Scientific Procedures) Act 1986 under the project number 70/8494. All protocols were approved by the local ethical committee at University of Birmingham.

### Crypt Isolation and Organoid Culture from Mouse Intestine.

Mouse organoids were generated from isolated small intestinal crypts as described previously (28). Further details are provided in *SI Appendix*.

### Cell Culture.

Details of culturing Caco-2 cells and HEK293T (human embryonic kidney 293T) cell lines and experiments involving these cells are provided in *SI Appendix*.

### Histology and Scoring.

Details of the preparation and processing of mouse tissues and organoids for histological analysis, immunohistochemistry, and scoring are described in *SI Appendix*.

### Organoid Growth Inhibition Assay.

Inhibitory effects of Lapatinib were measured using CellTiter-Glo 3D Cell Viability Assay (Promega) in a 96-well format. Mouse intestinal organoid crypts (∼25/well) were resuspended in 4% Matrigel/growth medium and plated in Matrigel-coated wells. An inhibitor was added after 24 h of culture in serial dilutions (10 nM, 25 nM, 100 nM, and 200 nM). The organoids were cultured for 6 d, and the cell viability was measured using CellTiter-Glo 3D Cell Viability Assay according to the manufacturer’s protocol. The luminescence was measured using the PHERAstar FS (BMG Labtech) microplate reader.

EVs quantification and depletion assays are described in *SI Appendix*.

### TGF-α Neutralizing Antibody Assay.

Tspan6 wild-type and Tspan6^−/−^ organoids were cultured in mouse intestinal organoid media without EGF in the presence of TGF-α neutralizing antibody (*SI Appendix*, Table S1) in serial dilutions (2.5, 5, 10, and 20 µg/mL) or control antibody (Rabbit IgG, Southern Biotech). Organoids were cultured for 7 d, and images were acquired using the Zeiss Axiovert 25 microscope equipped with Scion Corporation Greyscale camera, and images were analyzed using ImageJ.

### Enzyme-Linked Immunosorbent Assay.

To detect mouse amphiregulin and TGF-α in media conditioned by wild-type or Tspan6^−/−^ organoids, a sandwich-type enzyme-linked immunosorbent assay was used. Organoids were cultured for 3 d in mouse intestinal organoid medium with 50 ng/mL EGF or in the absence of EGF. The conditioned medium was collected and centrifuged at 1,000 × g for 10 min and analyzed according to manufacturers’ instructions (MyBioSource). The optical density was measured spectrophotometrically at a wavelength of 450 nm using the PHERAstar plate reader. The concentration of TGF-α was calculated using standard curve. Samples were run in duplicates.

### RNA Sequencing, Bioinformatics, and Statistical Analysis.

Details of RNA sequencing, bioinformatics, and statistical analyses are provided in *SI Appendix*.

## Supplementary Material

Supplementary File

## Data Availability

All study data are included in the article and/or *SI Appendix*.

## Change History

October 8, 2021: The license for this article has been updated.
