# Supplementary material for: Tetraspanin 6 is a regulator of carcinogenesis in colorectal cancer
Source: Proc Natl Acad Sci U S A. 2021 Sep 14;118(39):e2011411118. doi: 10.1073/pnas.2011411118 (PMC8488650; doi:10.1073/pnas.2011411118)
Supplement: Supplementary File [file pnas.2011411118.sapp.pdf]

Tetraspanin 6 (Tspan6) is a new regulator of carcinogenesis in colorectal cancer.

Regina Andrijes[1], Rahul K. Hejmadi[2], Matthew Pugh[1], Sundaresan Rajesh[1], Vera Novitskaya[1], Maha Ibrahim[1,4], Michael Overduin[1], Chris Tselepis[1], Gary W Middleton[1], Balázs Győrffy[3], Andrew D Beggs*[1], Fedor Berditchevski*[1§]

Running title: Tetraspanin 6 in colorectal cancer

[1]Institute of Cancer and Genomic Sciences, The University of Birmingham, Edgbaston, Birmingham, UK, [2]University Hospitals Birmingham NHSFT, Birmingham, UK, [3]Department of Bioinformatics, Semmelweis University, Budapest, Hungary; 2nd Dept. of Pediatrics, Semmelweis University, Budapest, Hungary; TTK Cancer Biomarker Research Group, Budapest, Hungary, [4]South Egypt Cancer Institute, Assiut University, Assiut 71515, Egypt.

[§]Corresponding authors: Institute of Cancer and Genomic Sciences, The University of Birmingham, Edgbaston, Birmingham, B15 2TT, United Kingdom; Email: f.berditchevski@bham.ac.uk;

*** = contributed equally to the study**

SUPPORTING INFORMATION

Antibodies. All antibodies used in this study are listed in the Supporting Table1

Crypt isolation and organoid culture from mouse intestine. Crypts were counted and resuspended in Matrigel (BD Biosciences). A total of 200 crypts were embedded into 50 µl of Matrigel (BD Bioscience) and plated in 24-well plates. After polymerization of Matrigel, 500 µl of Intesticult™ organoid growth medium (StemCell Technologies) was added. Media was changed every 2 days. For passage, organoids were mechanically dissociated into single-crypt domains and transferred into fresh Matrigel. Passage was performed every 1-2 weeks with a 1:5 split ratio. For co-culture and growth factor withdrawal experiments organoids were cultured in organoid basal media (Advanced DMEM/F-12 (Gibco), 1xB27 (ThermoFisher Scientific), 1xN2 (ThermoFisher Scientific), 1.25 mM N-Acetyl Cysteine (Sigma- Aldrich), 10mM Nicotinamide (Sigma-Aldrich), 100µg/ml Primocin (Vivogen)) supplemented with 100 ng/ml Noggin (Peprotech), and/or 20% (v/v) R-Spondin-1 conditioned medium, and/or 50 ng/ml EGF (Peprotech). R-spondin-1 (Rspo1)- conditioned medium was generated using manufacturer’s protocol from HA- Rspondin-1-Fc stably transfected HEK293T cells (Amsbio).

Cell culture

Caco-2 cells and HEK293T cells were purchased from American Type Culture Collection (ATCC). All cells were cultured in DMEM (Sigma-Aldrich) supplemented with 10% heat inactivated fetal bovine serum (Gibco). Caco-2 cells were authenticated by Short Tandem Repeat using GenePrint® 10 System (Promega). All cell lines have been tested for mycoplasma contamination using Mycoalert® Mycoplasma Detection Kit (Lonza) and were confirmed mycoplasma-free. For 3D culture, Caco-2 were seeded on plates coated with growth factor reduced Matrigel™ (BD Bioscience) and cultured in complete growth medium supplemented with 0.4 mg/ml Matrigel™. To analyse lumenogenesis in Caco-2 colonies, images of 25 fields were taken using phase-contrast microscopy (Nikon). The diameter of colonies and lumens were measured using ImageJ Software. Approximately 100 colonies per condition were analysed. For the experiments with cetuximab, cells were plated on Matrigel™-coated LabTek in 0.4 mg/ml Matrigel™ in complete growth medium. After 24 hours 25 µg/ml cetuximab was added. For the syntenin-1

siRNA knockdown, Caco-2 cells were reverse-transfected with control siRNA or siRNA targeting syntenin-1 (smart pool of four siRNAs: GGAGAGAAGAUUACCAUGA, GACCAAGUACUUCAGAUCA, GGAUGGUCUUAGAAUAUUU, GCAUUUGACUCUUAAGAUU, Dharmacon, Lafayette, CO, USA). After 24 hours, transfected cells were plated Matrigel™- coated LabTek in 0.4 mg/ml Matrigel™ in complete growth medium and cultured for 5 days.

Stable gene expression cell line establishment

Stable expression of FLAG-Tspan6 in Caco-2 cells was established using lentiviral transduction. The vectors psPAX2 and pMD2.G were constructed and provided by D. Trono (Geneva, Switzerland); the vector pLVx-puro is used to subclone the gene of interest. Lentiviral particles were produced using PEI-based (polyethyleimine) transfection protocol on 70% confluent HEK293T. HEK293T cells were transfected with a plasmid mix containing the vector pLVx encoding FLAG-Tspan6, the packaging construct psPAX2 and the envelope plasmid pMD2G-VSVG at the ratio 1.5:2:1. 48-hour post-transfection virus containing supernatant was harvested and supplemented with polybrene (1µg/ml) and added to CaCo-2. For stable gene transduction, selection with 2 µg/ml puromycin started 48 hours after the last infection cycle. Transfection efficiency was determined by Western blot analysis using polyclonal anti-Tspan6 antibody (Thermo Fisher Scientific) and monoclonal anti-FLAG antibody (Sigma-Aldrich).

Western blot. For western blot analysis whole cell lysates were prepared in 1x Laemmli (63 mM Tris-HCl pH 6.8 2.1 ml, 10% v/v glycerol, 2% w/v SDS) supplemented with 1x Protease/Phosphatase Inhibitor Cocktail (Cell Signalling Technologies). Whole cell extracts were fractionated by SDS-PAGE and transferred to a nitrocellulose membrane. Membranes were incubated with 5% non-fat milk in TBST (10 mMTris, pH 8.0, 150 mM NaCl, 0.5% Tween 20) for 60 min, the membrane was washed with TBST and incubated overnight at 4 °C with an appropriate primary antibody diluted in 3% w/v BSA-TBST-3 mM NaN_3 (all antibodies used in this study are listed in the *SI Appendix,* Table1). After washing 3 times with TBST for 5 minutes, membranes were incubated with secondary antibody conjugated with fluorescent dye, goat anti-mouse Ab IRDye 680LT, or goat anti-rabbit Ab IRDye 800CW (LI-COR), diluted in 5% (w/v) non-fat milk in TBST for 1 hour at room temperature.

Then, membranes were visualized using Odyssey®CLx Infrared Imaging System (LI-COR).

Co-immunoprecipitation (co-IP) assay. For co-immunoprecipitation studies, 3x10^6 cells were cultured in 10cm tissue culture dish (Corning) until 90% confluent. Subsequently, the monolayer of cells was washed with sterile PBS and proteins were solubilised in 500 µl of protein extraction solution (0.8% Brij98-0.2% Triton-X- 100-PBS) for 4hr at 4°C. Subsequently, supernatants were immunoprecipitated with 20 µl of ANTI-FLAG M2 agarose beads (Sigma-Aldrich) 4hr at 4°C. Subsequently, beads were washed 3x with ice-cold PBS and retained proteins were eluted by 30 µl of 1x Laemmli buffer and boiling for 10 minutes at 60°C. For normalized co-IP blots, proteins were extracted from equal number of cells for each cell line.

Histology and scoring

Mouse intestinal tissue and organoids were fixed in 4% paraformaldehyde followed by dehydration, paraffin embedding, sectioning and standard H&E staining. For immunohistochemical analyses, human and mouse formalin fixed paraffin embedded (FFPE) tissues were sectioned in 4µm sections. Slides were deparaffinized and antigen retrieval was performed with citric acid buffer (pH 6) or EDTA (pH 9.0, Sigma-Aldrich) using a microwave. Endogenous peroxidase was inhibited with 0.3% hydrogen peroxide. Sections were blocked with 5% casein (Vecor Labs) then washed with sterile TBS (pH 7.5) before overnight incubation at 4°C with the appropriate primary antibodies (EV Table1). After slides were washed with TBS and incubated in secondary antibody (1:100 biotinylated goat anti-rabbit IgG or rabbit anti-goat IgG (Vector Laboratories) for 30 min at room temperature. After washing with TBS, slides were incubated in diaminobenzidine tetrahydrochloride (DAB) solution (Vector Laboratories) for up to two minutes, depending on the primary antibody. After washing in distilled water, the sections were counterstained with haematoxylin (Vector Laboratories), dehydrated through ethanol and xylene, and cover-slipped using a xylene-based mounting medium (Fisher Scientific). For the blind test, sections were randomized and analysed by two independent pathologists. Intestinal neoplasia was assessed according to the 2010 Mouse Models of Human Cancers Consortium criteria (1) taking into account the model specific pathological features of $APC^{min/+}$ mice (2). Where these criteria were not applicable, the 2019 WHO Classification of Tumours of the Digestive System classification was employed (3). Tspan6 staining of CRC was scored by two independent pathologists in a blind test. H-score was determined by evaluation of the intensity of membranous staining (1 - weak or trace; 2 - moderate; 3 – intense) and relative abundance of present staining (1 – 1-33.3%; 2 – 33.4%-66.6%;

3 – 66.7%-100% of analysed section). The final H-score was determined by adding the results of multiplication of the percentage of cells with staining intensity ordinal value.

Immunofluorescence. Mouse intestinal organoids were cultured in 2% Matrigel™ on pre-coated with 100% Matrigel 8-well Nunc® Lab-Tek® Chamber Slide™ system for 5 days. Cells were fixed with 4% paraformaldehyde/3% sucrose/PBS supplemented with 1mM $CaCl_2$ and 0.5mM $MgCl_2$ for 20 min at room temperature and permeabilized with 0.1% (v/v) Triton X-100 for 5 minutes. Organoids were incubated with blocking buffer (10% heat inactivated goat serum/PBS) for 1 hour at room temperature. Subsequently, cells were incubated with primary antibodies overnight at 4°C. On the following day, organoids were incubated with fluorophore-conjugated secondary antibodies and/or Phalloidin Alexa Fluor 568 (Molecular Probes) (diluted 1:40 in 1%BSA/PBS) for 1 hour at room temperature. Cells were then counterstained with Hoechst33342 (1µg/ml in PBS) for 5 min at room temperature. Washes between each step were performed with 1%BSA/PBS (3x 10 min). The images were captured using Zeiss LSM780 confocal system with 40X oil immersion objective.

Extracellular vesicle depletion assay

Tspan6$^{-/-}$ organoids were cultured in mouse intestinal organoid media without EGF. After three days, conditioned media was collected and divided into two fractions. Extracellular vesicles (EVs) were depleted from one fraction by multi-step centrifugation (4). The supernatant and fraction II of conditioned medium were supplemented with 20% (v/v) R-Spondin-1 conditioned medium, 100 ng/ml Noggin (Peprotech), 1x B27 (ThermoFisher Scientific), 1x N2 (ThermoFisher Scientific), 1.25 mM N-Acetyl Cysteine (Sigma-Aldrich), 10 mM Nicotinamide (Sigma-Aldrich)

and used to culture wild-type or Tspan6$^{-/-}$ organoids. Fresh conditioned media and EVs depleted conditioned medium were prepared for media change each three days of culture. The organoids were cultured in EVs depleted medium for 7 days.

Extracellular vesicle quantification

Mouse intestinal organoids were cultured for 6 days in mouse intestinal organoids media Intesticult™ (STEMCell Technologies) or in mouse basal organoid media supplemented with growth factors without EGF ligands. The conditioned media was collected and filtered using 0.45 µm sterile filters and loaded into the cell. Extracellular vesicle size and concentration were assessed by NTA, using Nanosight LM10 v3.3.104 (Malvern Technologies) equipped with a sample chamber and a 532 nm laser. The samples were injected into the sample chamber with sterile syringes. For each measurement, five 1-min videos were captured under the following conditions at 25°C. After capture, the videos have been analysed by the in-build NanoSight Software NTA 3.3 Build 3.3.104 with a detection threshold of 5.

NMR analysis

For the heteronuclear magnetic resonance spectroscopy (NMR) analysis of syntenin-1 and Tspan6 interaction, uniformly ^{15}N-labeled syntenin-1 PDZ tandem domain (113 to 273), referred to as "syntenin-1 PDZ1-2," was expressed as a GST fusion in *Escherichia coli* BL21 (DE3) at 25 °C in M9 minimal medium using $^{15}NH_4Cl$ as the sole nitrogen source and purified as as reported previously (5). The NMR samples contained 200 µM protein, 150 mM NaCl, 500 µM TCEP [Tris(2-carboxyethyl)phosphine], and 50 µM AEBSF in 50 mM Tris buffer (pH 7.5). All NMR spectra were recorded and data were processed as reported previously (2). Synthesized 12-mer C-terminal TGFα and Tspan6 peptides was purchased from Sigma-Genosys. Titrations of syntenin-1 PDZ1-2 protein with C-terminal TGFα peptide were conducted by recording a series of ^{1}H,^{15}N HSQC spectra of ^{15}N- labeled protein (200 µM), with increasing molar concentrations of the peptide ligand, up to a protein-to-peptide ratio of 1:8. The combined backbone ^{1}H and ^{15}N chemical shift changes, $\Delta\delta$, were calculated as reported earlier (5). To this reaction mixture (Syntenin-1 PDZ1-2 + TGFα) was added 8-fold molar excess (1.6 mM final) of Tspan6 C-terminal peptide and the NMR spectra was recorded. The $\Delta\delta$ values were calculated as described before.

RNA sequencing and analysis

RNA samples were extracted from intestinal polyps of APC$^{min/+}$ (n=3) and APC$^{min/+}$Tspan6$^{-/-}$ (n=5) mice using RNeasy RNA extraction kit (Qiagen). The quality of

RNAs were checked using Agilent 2200 TapeStation (Agilent Technologies) and a total of 5-100 ng RNA from each sample with RNA Integrity Number (RIN) of >7.0 was used for library preparation. Poly-adenylated RNA libraries were prepared using the automated Neoprep Library System (Illumina) according to manufacturer's protocol. The libraries were sequenced on an Illumina NextSeq 550 (Illumina) using a High out-put flow cell 150 cycle (cat. No. FC-404-2002) and a paired end 75 cycle program. The aim was to achieve at least 25 million reads per library. The RNAseq reads were aligned to the mouse genome (Mus musculus mm10 Refseq) using the STAR Aligner v.2.5. Sequencing reads were aligned to GRCm38 mouse genome with STAR aligner (v2.5.2b). Reads mapping to genes were counted by the same software. Normalisation of read counts and differential expression analysis between the control and the TSPAN6 knocked-out samples was performed with the Partek EM algorithm. Pathway analysis was carried out using the KEGG pathway analysis module of Partek Flow.

Statistical analyses

Statistical analysis of data from all studies was performed using Graph Pad Prism v7.04 (Graphpad Software Inc.) and presented as the mean with standard error mean (SEM) or standard deviation and indicated in figure legends. For box-and-whisker plots, data presented as the median with highest and lowest values. To analyse normally distributed data from two unpaired groups a Student t-test was utilised. For analysis of unpaired non-parametric data a Mann-Whitney U test was used. To compare multiple unmatched groups one-way ANOVA test was used. P value $p< 0.05$ was considered as statistically significant. Asterisks indicate levels of significance (* – $p < 0.05$; ** – $p < 0.01$; *** – $p < 0.001$). For multivariate logistic regression, all analyses were carried out in Stata 16.1

Supporting Figures

Fig S1

Western blot showing the decrease in pEGFR in $APC^{min/+}$$Tspan6^{-/-}$ mouse intestinal organoids treated with different concentrations of lapatinib. In control experiments organoids were incubated with DMSO. Western blot with anti-actin mAb antibody shows equal loading of the samples.

Fig S2.

Tspan6 expression promotes lumenogenesis of Caco-2 in 3-D ECM by suppressing autocrine EGFR-dependent signalling. *(A)* Representative images of Caco-2 cells grown in 3-D ECM in complete media (control) or in media supplemented with 25 µg/ml cetuximab (+CTX). The graph represents the quantification of lumen-bearing colonies and colonies without lumen in the absence (control) and the presence (+CTX) of 25 µg/ml cetuximab. Data is presented as mean ± SEM, ***P*=0.0071, ****P*=0.0002, n=3. Scale bars: 50 µm. *(B)* Western blot showing expression of Tspan6 in Caco-2/pLVx (control) and CaCo-2/Tspan6 cells; representative images of colonies formed by Caco-2/pLVx and CaCo-2/Tspan6 cells cultured in 3-D ECM. The graph represents quantification of colonies with and without lumen formed by Caco-2/pLVx and CaCo-2/Tspan6 cells cultured in 3-D ECM. Data is presented as mean □ SEM, ****P*=0.0009, n=3. Scale bars: 50 µm. *(C)* Quantification of EGFR activation (pEGFR) relative to the total level of EGFR expression in Caco-2/pLVx and CaCo-2/Tspan6 cells cultured in 3-D ECM. Data presented as mean of 2 independent experiments □ SEM. Data is presented as mean □ SEM, *P*=0.0079.

Fig S3.

Mouse intestinal organoids undergo apoptosis in the absence of EGF from the complete growth media. *(A)* Representative images of live and dead mouse intestinal organoids. Scale bars: 100 µm. (B) Immunofluorescent labelling of cleaved caspase-3 in $APC^{min/+}$ mouse intestinal organoids cultured in complete growth media (control) or in the absence of EGF (-EGF) for 5 days. Scale bars: 20 µm.

Fig S4.

Tspan6 loss results in EGF-independent growth of intestinal organoids derived from WT and Tspan6$^{-/-}$ mice. *(A)* Tspan6-mediated MAPK activation is EGFR-dependent. Representative images of WT and Tspan6$^{-/-}$ organoids in response to increasing concentrations of pan-EGFR inhibitor lapatinib (LPT) after 6 days of culture. Scale bars: 100 μm. *(B)* A dose-response curve of WT and Tspan6$^{-/-}$ mouse intestinal organoids treated with lapatinib. Cell viability was measured by CellTiter-Glo3.0 ATP-based assay after 6 days of treatment. Plots were normalised to DMSO (control) treated organoids. *(C)* Representative images of WT and Tspan6$^{-/-}$ organoids in response to increasing concentrations of MEK inhibitor U0126 after 6 days of culture. Scale bars: 100 μm.

Fig S5.

Tspan6 expression does not affect EV secretion and expression of syntenin-1. *(A)* Representative NTA profiles of total EVs derived from WT (black) and Tspan6$^{-/-}$ (red) organoids respectively cultured for 5 days in -EGF media. Total concentration of vesicles SEM are also indicated on the graph, with 1.75x10^6 +/-8.47x10^3 vesicles/ml and 1.33x10^6 +/-6.00x10^3 vesicles/ml respectively. *(B-C)* Western blot representing the expression of syntenin-1 in organoids derived from WT, Tspan6$^{-/-}$, APC$^{min/+}$ and APC$^{min/+}$Tspan6$^{-/-}$ mice *(B)* and Caco-2 cells line *(C)* expressing empty vector (pLVx) or Flag-Tspan6 (Tspan6). (*D-E)* Graphs represent the densitometry analysis of syntenin-1 expression in organoids *(D)* and Caco-2 cells *(E)*. The expression was normalised to β-tubulin levels (n=2, n=4 respectively). Data presented as mean SEM. ns – not significant (One-way ANOVA test).

Fig S6.

Tspan6 is associated with TGFα in Caco-2 cells. Immunoprecipitation of exogenously expressed FLAG-Tspan6 with endogenous TGFα from Caco-2 cells. Mouse isotype control antibody IgG1 was used as a negative control.

Fig S7.

Knockdown of syntenin-1 results in reduction of lumen formation in colonies of Caco-2 cells in 3D-ECM. *(A)* Western blot showing level of syntenin-1 expression in Caco-2 cells treated with control siRNA and syntenin-1 siRNA for 5 days. *(B)* Representative images of Caco-2 cells transfected with siRNA (control or syntenin-1) and cultured for 5 days in 3D-ECM after 24 hours of transfection. Scale bars: 50 μm. *(C)* Quantification

of Caco-2 colonies with lumen when treated with control (siCtrl) and syntenin-1 siRNA (siSyntenin-1). Data presented as mean ± SEM, $^{**}P = 0.0089$ (n=2).

Fig S8.

The decreased expression of Tspan6 correlates with advanced tumour stages and lymphovascular invasion.*(A-C)* Kaplan–Meier survival curves for overall survival of CRC patients with adenocarcinoma with Tspan6-high and Tspan6-low expression in tumours with *(A)* lymphovascular invasion (LVI), *(B)* metastasis (M1), and *(C)* lymph node metastasis (N2). *P* values were determined by log-rank test and are shown for comparisons of Tspan6-high expression.

1. M. K. Washington *et al.*, Pathology of rodent models of intestinal cancer: progress report and recommendations. *Gastroenterology* **144**, 705-717 (2013).
2. K. Washington, Zemper, A.E.D., Apc-related models of intestinal neoplasia: a brief review for pathologists. *Surg.Exp.Pathol* **2**, https://doi.org/10.1186/s42047-42019-40036-42049 (2019).
3. I. D. Nagtegaal *et al.*, The 2019 WHO classification of tumours of the digestive system. *Histopathology* **76**, 182-188 (2020).
4. Thery, S. Amigorena, G. Raposo, A. Clayton, Isolation and characterization of exosomes from cell culture supernatants and biological fluids. *Current protocols in cell biology* **Chapter 3**, Unit 3 22 (2006).
5. N. Latysheva *et al.*, Syntenin-1 is a new component of tetraspanin-enriched microdomains: mechanisms and consequences of the interaction of syntenin-1 with CD63. *Mol. Cell Biol.* **26**, 7707-7718 (2006).

Figure S1

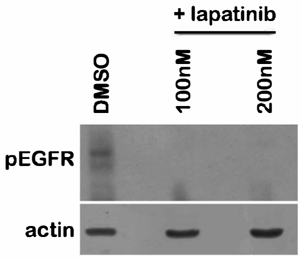

Figure S2

A

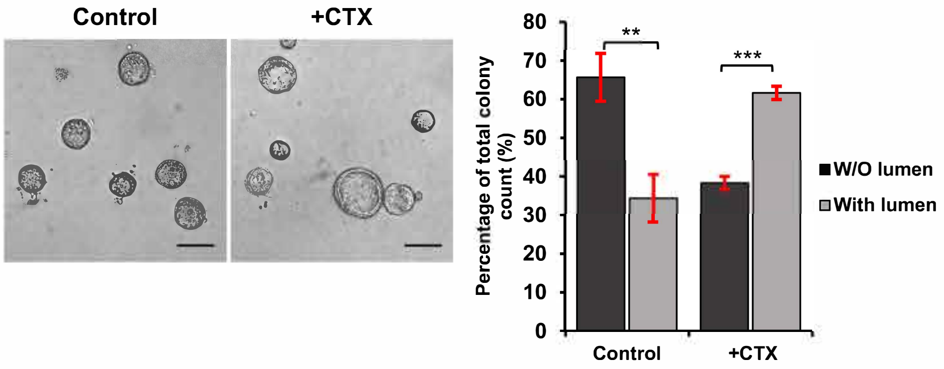

B

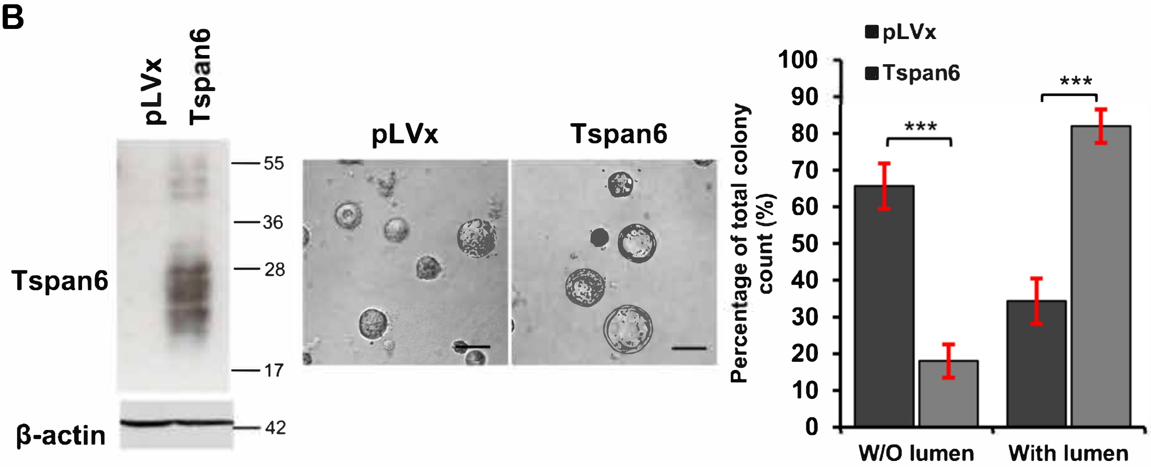

C

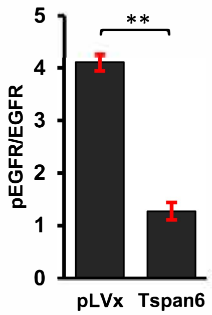

Figure S3

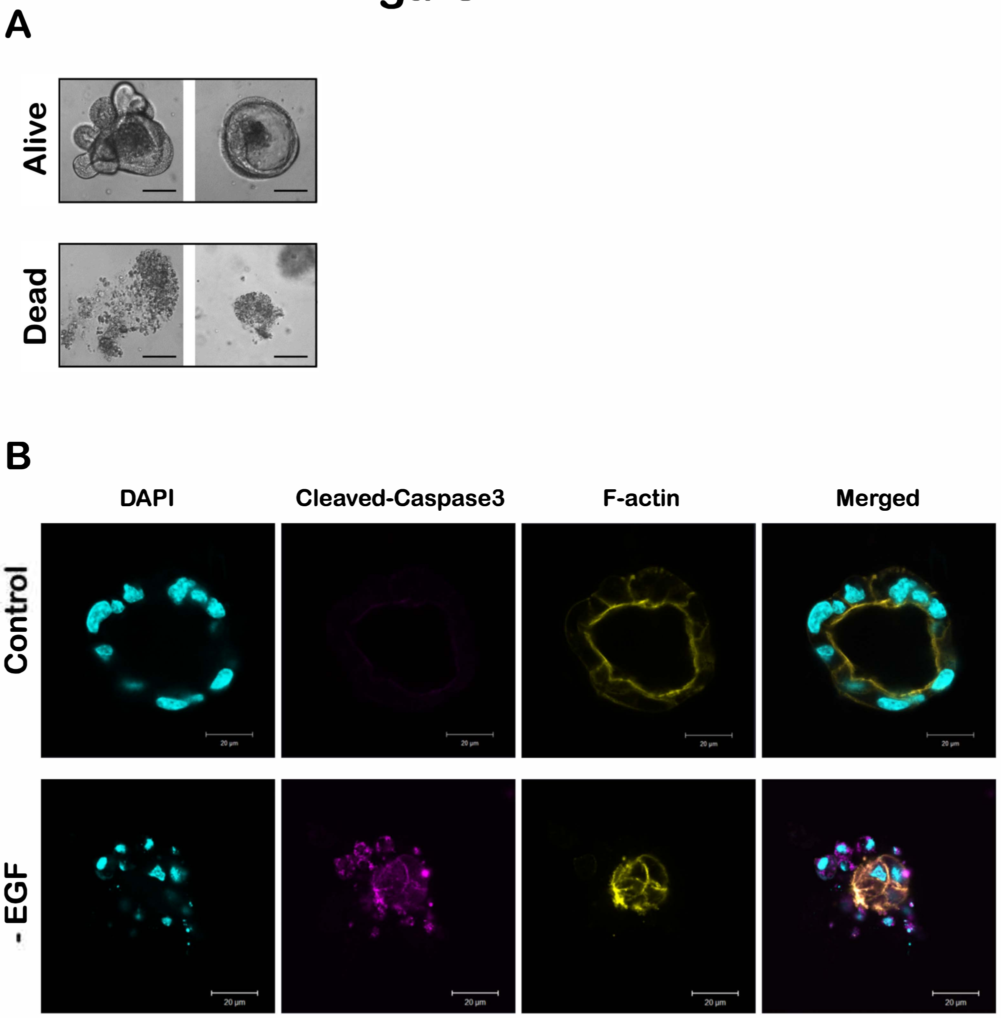
A
Alive
Dead
B
DAPI
Cleaved-Caspase3
F-actin
Merged
Control
- EGF
20 µm

Figure S4

A

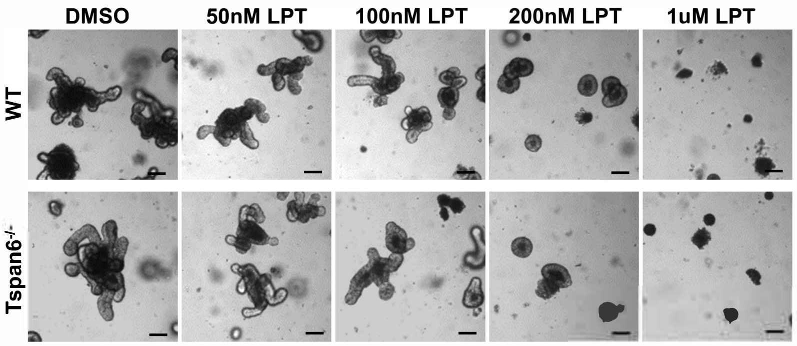

B

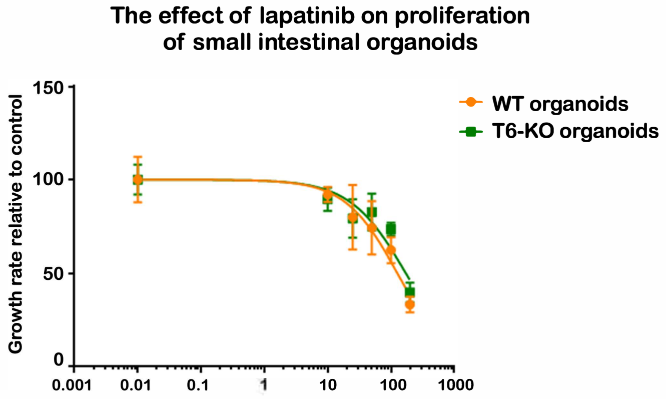

C

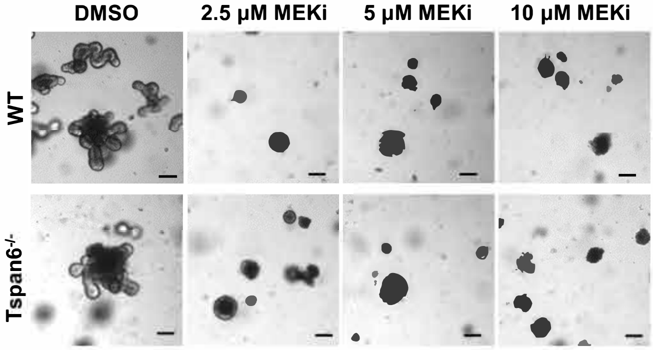

Figure S5

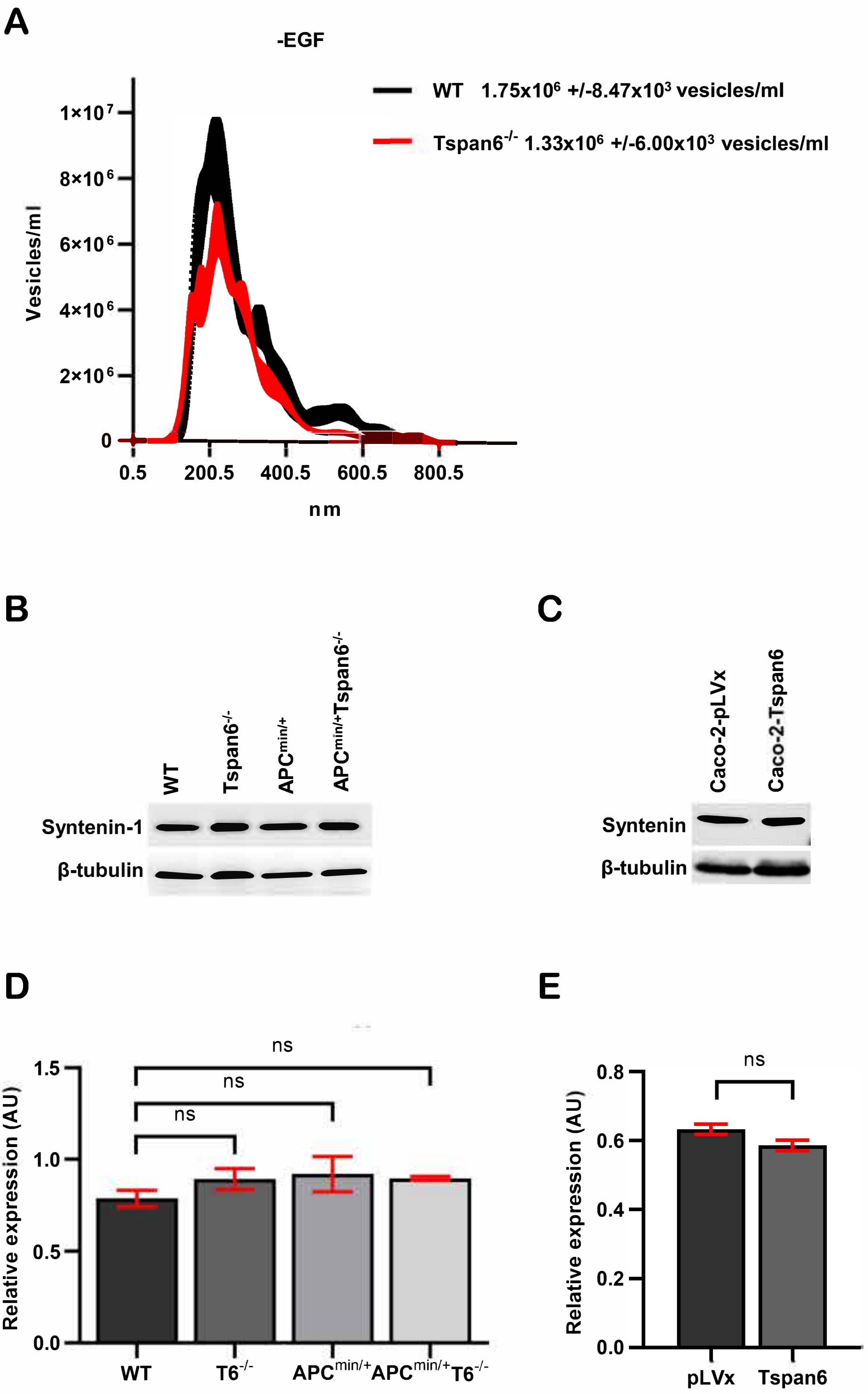

A
-EGF
WT 1.75x10⁶ +/-8.47x10³ vesicles/ml
Tspan6-/- 1.33x10⁶ +/-6.00x10³ vesicles/ml
Vesicles/ml
1×10⁷
8×10⁶
6×10⁶
4×10⁶
2×10⁶
0
0.5
200.5
400.5
600.5
800.5
nm
B
WT
Tspan6-/-
APCmin/+
APCmin/+Tspan6-/-
Syntenin-1
β-tubulin
C
Caco-2-pLVx
Caco-2-Tspan6
Syntenin
β-tubulin
D
Relative expression (AU)
1.5
1.0
0.5
0.0
ns
ns
ns
WT
T6-/-
APCmin/+
APCmin/+T6-/-
E
Relative expression (AU)
0.8
0.6
0.4
0.2
0.0
ns
pLVx
Tspan6

Figure S6

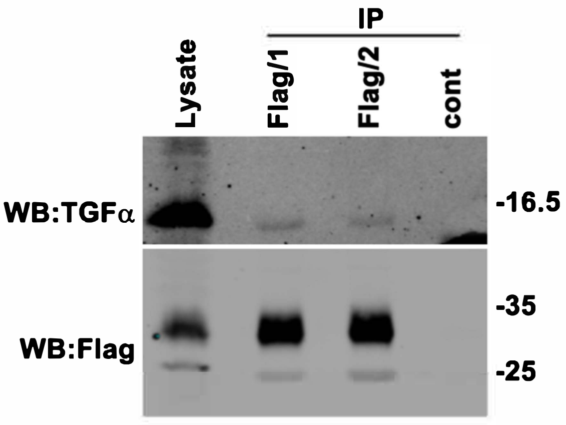

Figure S7

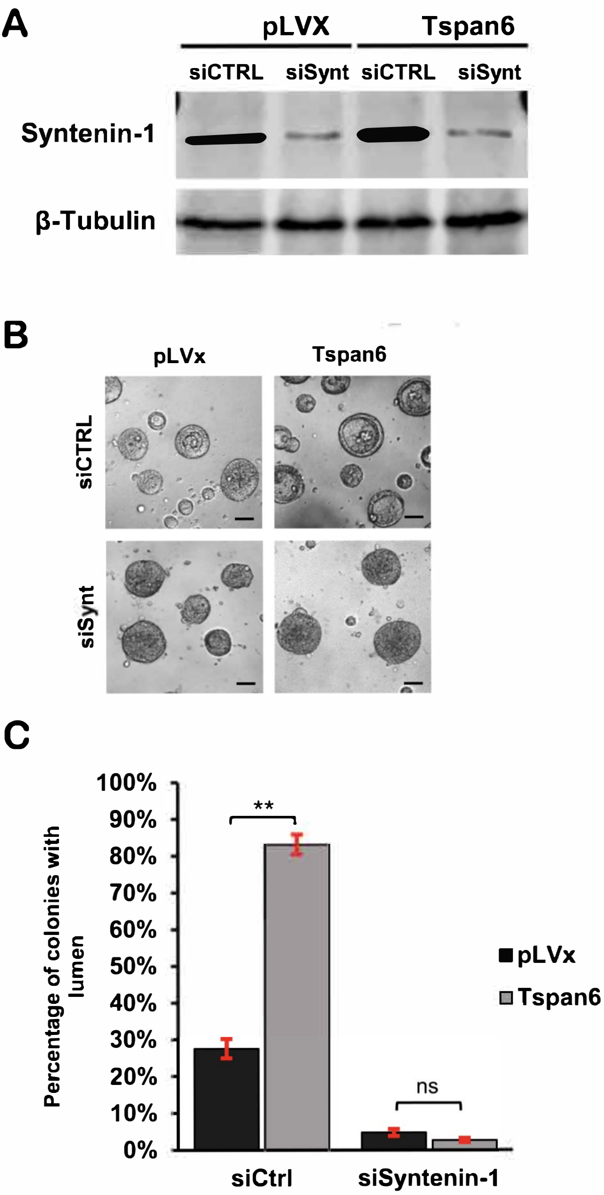
A
pLVX
Tspan6
siCTRL
siSynt
siCTRL
siSynt
Syntenin-1
β-Tubulin
B
pLVx
Tspan6
siCTRL
siSynt
C
Percentage of colonies with lumen
100%
90%
80%
70%
60%
50%
40%
30%
20%
10%
0%
**
ns
siCtrl
siSyntenin-1
pLVx
Tspan6

Figure S8

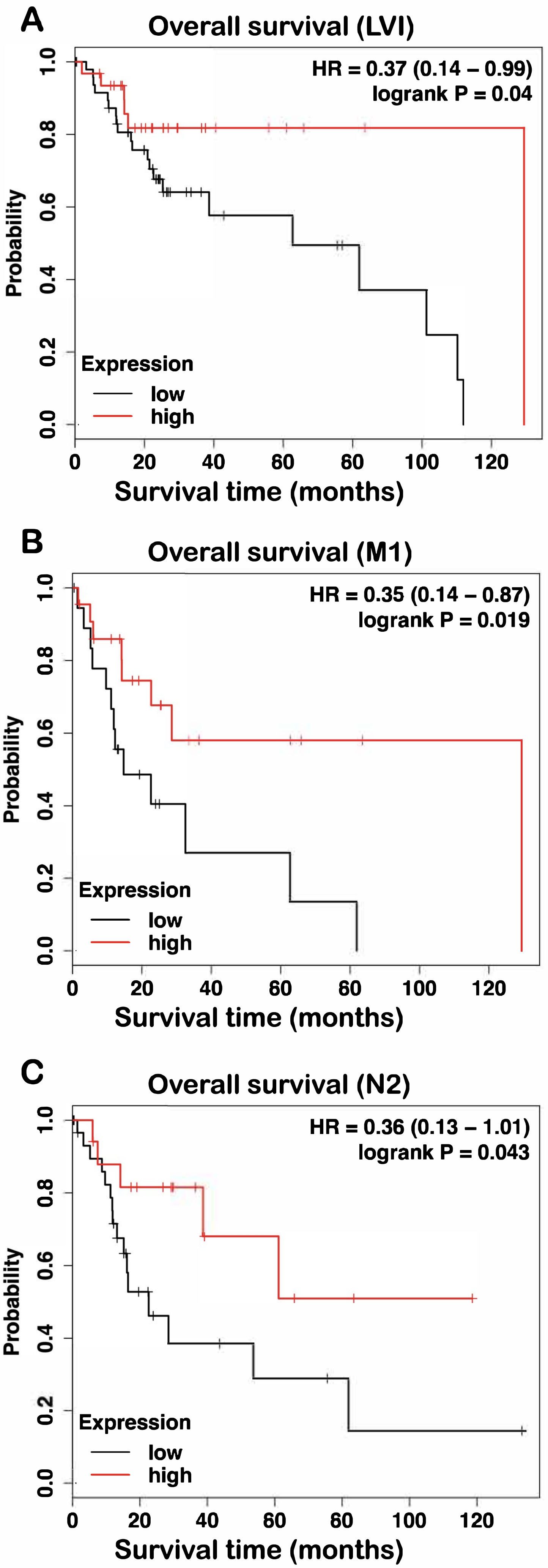
A
Overall survival (LVI)
HR = 0.37 (0.14 – 0.99)
logrank P = 0.04
Expression
low
high
Probability
0.0 0.2 0.4 0.6 0.8 1.0
0 20 40 60 60 100 120
Survival time (months)
B
Overall survival (M1)
HR = 0.35 (0.14 – 0.87)
logrank P = 0.019
Expression
low
high
Probability
0.0 0.2 0.4 0.6 0.8 1.0
0 20 40 60 60 100 120
Survival time (months)
C
Overall survival (N2)
HR = 0.36 (0.13 – 1.01)
logrank P = 0.043
Expression
low
high
Probability
0.0 0.2 0.4 0.6 0.8 1.0
0 20 40 60 60 100 120
Survival time (months)

Table S1. Average weight and size of age-matched wild type, Tspan6$^{-/-}$, APC$^{min/+}$ and APC^{min}Tspan6$^{-/-}$ mice (WT n=7, Tspan6$^{-/-}$ n=16, APC$^{min/+}$ n=17, APC$^{min/+}$Tspan6$^{-/-}$ n=36) and their organs.

	WT (n=7)	Tspan6$^{-/-}$ (n=16)	p-value (WT vs. Tspan6$^{-/-}$)	APC$^{min/+}$ (n=17)	APC$^{min/+}$Tspan6$^{-/-}$ (n=36)	p-value (APC$^{min/+}$ vs. APC$^{min/+}$Tspan6$^{-/-}$)
**Average mouse weigh (g)**	30.89	34.48	**0.049**	22.21	24.37	**0.045**
**Average body length (cm)**	9.67	10.19	**0.115**	9	9.23	**0.003**
**Length of Intestine (cm)**	43.7	45.78	**0.332**	47.11	45.63	**0.261**
**Heart (g)**	0.21	0.21	**0.850**	0.19	0.19	**0.912**
**Liver (g)**	1.79	1.89	**0.946**	1.44	1.45	**0.356**
**Lungs (g)**	0.17	0.2	**0.098**	0.15	0.18	**0.147**
**Pancreas (g)**	0.17	0.2	**0.384**	0.15	0.17	**0.263**
**Spleen (g)**	0.03	0.04	**0.822**	0.34	0.34	**0.267**
**Thyroid (g)**	0.19	0.23	**0.863**	0.13	0.13	**0.037**
**Kidneys (g)**	0.43	0.46	**0.216**	0.28	0.31	**0.320**
